# Diagnostic Accuracy of Molecular Testing on Saliva and Oral Swabs for Pulmonary Tuberculosis

**DOI:** 10.1093/cid/ciag055

**Published:** 2026-03-12

**Authors:** Deninson Alejandro Vargas, Jose Fernando Fuertes-Bucheli, Andrea Sanchez-Hidalgo, Jairo Palomares Velosa, Alvaro Mauricio Lasso, Amanda J Gupta, Alvaro J Martinez-Valencia, Gustavo Díaz, Lucy Luna, Neal Alexander, Beatriz Eugenia Ferro, J Lucian Davis

**Affiliations:** Centro Internacional de Entrenamiento e Investigaciones Médicas (CIDEIM), Cali, Colombia; Universidad Icesi, Cali, Colombia; Centro Internacional de Entrenamiento e Investigaciones Médicas (CIDEIM), Cali, Colombia; Departamento de Ciencias Básicas Médicas, Facultad de Ciencias de la Salud, Universidad Icesi, Cali, Colombia; Centro Internacional de Entrenamiento e Investigaciones Médicas (CIDEIM), Cali, Colombia; Centro Internacional de Entrenamiento e Investigaciones Médicas (CIDEIM), Cali, Colombia; Centro Internacional de Entrenamiento e Investigaciones Médicas (CIDEIM), Cali, Colombia; Universidad Icesi, Cali, Colombia; Department of Epidemiology of Microbial Diseases, Yale School of Public Health, New Haven, Connecticut, USA; Johns Hopkins Bloomberg School of Public Health, Baltimore, Maryland, USA; Centro Internacional de Entrenamiento e Investigaciones Médicas (CIDEIM), Cali, Colombia; Universidad Icesi, Cali, Colombia; Centro de Investigaciones Clínicas, Fundación Valle del Lili, Cali, Colombia; Servicio de Enfermedades Infecciosas, Fundación Valle del Lili, Cali, Colombia; Centro Internacional de Entrenamiento e Investigaciones Médicas (CIDEIM), Cali, Colombia; Programa de TB y VIH, Secretaría de Salud Distrital, Cali, Colombia; Centro Internacional de Entrenamiento e Investigaciones Médicas (CIDEIM), Cali, Colombia; Universidad Icesi, Cali, Colombia; Departamento de Ciencias Básicas Médicas, Facultad de Ciencias de la Salud, Universidad Icesi, Cali, Colombia; Department of Epidemiology of Microbial Diseases, Yale School of Public Health, New Haven, Connecticut, USA; Section of Pulmonary, Critical Care, and Sleep Medicine, Yale School of Medicine, New Haven, Connecticut, USA

**Keywords:** tuberculosis, *Mycobacterium tuberculosis*, molecular diagnostic techniques, sensitivity and specificity, Colombia

## Abstract

**Background:**

Rapid, accurate, nonsputum tests are needed to close gaps in tuberculosis (TB) detection. We evaluated the diagnostic performance of molecular testing on saliva and oral swabs.

**Methods:**

We conducted a nested case–control study with 1:1 incidence-density sampling within a prospective cohort of adults and children undergoing evaluation for pulmonary TB at primary care centers in Colombia (July 2023–August 2024). Participants provided a sputum sample for liquid mycobacterial culture and paired saliva and nylon-flocked oral swabs for storage at −80°C. A microbiologist blinded to clinical and culture data performed Xpert MTB/RIF Ultra on thawed saliva and on swab eluate, each mixed 1:1 with sample reagent. We calculated the sensitivity and specificity of saliva and swab against sputum culture and compared them using McNemar's test.

**Results:**

Among 648 enrolled participants, we tested saliva and swabs from all 95 individuals with culture-confirmed TB and 95 matched culture-negative controls (n = 190). Saliva sensitivity was 90.5% (95% confidence interval [CI], 82.8–95.6), and specificity was 95.8% (95% CI, 89.6–98.8). Swab sensitivity was 71.6% (95% CI, 61.4–80.4), and specificity was 99% (95% CI, 94.3–100). Saliva sensitivity exceeded that of swab by an absolute difference of 18.9% (95% CI, +10.0 to +27.9, *P* < .001), but there was no significant difference in specificity (−3.2%, 95% CI, −7.7 to +1.4, *P* = .25). Over 95% of participants found saliva and swab collection procedures acceptable.

**Conclusions:**

Both saliva and swabs were highly sensitive and specific for culture-confirmed pulmonary TB. Saliva sensitivity exceeded the World Health Organization's ≥80% target for a low-complexity, nonsputum TB diagnostic test.

## Teaser

Molecular testing of saliva was more sensitive but less specific for pulmonary tuberculosis (TB) diagnosis than oral swabs. Saliva offers a promising specimen for noninvasive TB diagnosis, approaching the World Health Organization's minimal requirements for a nonsputum TB diagnostic test.

Approximately, one-quarter of the 10.8 million persons with tuberculosis (TB) worldwide in 2023 went undetected. Among those diagnosed with pulmonary TB and reported to public health authorities, only 62% were microbiologically confirmed, and only 48% underwent initial testing with a World Health Organization (WHO)-recommended rapid diagnostic test [1]. Although sputum is the standard specimen for diagnosing pulmonary TB, it can be difficult to collect from certain populations, including children, people with HIV (PWH), those who are asymptomatic, and those with a nonproductive cough [2]. Another disadvantage of sputum collection is that it requires strict adherence to airborne infection control measures to protect bystanders, including health workers.

Nonsputum samples collected from the oral cavity offer a potentially more feasible alternative for TB diagnosis because they are minimally invasive and often simpler, more comfortable, and possibly safer to collect than sputum. If a molecular or other rapid diagnostic test is performed on these specimens, these samples could also improve the feasibility and diagnostic yield of active case-finding initiatives in clinic and community settings and increase the proportion of persons with TB receiving microbiologically confirmed diagnoses [2–4]. World Health Organization has recommended minimum diagnostic performance targets for nonsputum TB diagnostic tests of ≥80% sensitivity and ≥98% specificity [5, 6]. The reported sensitivity of the Xpert MTB/RIF Ultra (Xpert-Ultra) molecular assay ranges from 45% to 78% on swabs [7], 71% to 80% on oral washes [8], and 79% to 90% on saliva [9, 10], with lower sensitivity in PWH and children across all sample types [7–11]. No studies have directly compared the 2 most promising minimally invasive methods, saliva and swabs, in the same population [7–11].

Accurate nonsputum diagnostics for TB is a global priority to end TB as a public health threat [2, 12, 13]. To evaluate the diagnostic performance of saliva and oral swabs tested with molecular assays, we conducted a prospective observational study in Cali, Colombia.

## METHODS

### Study Design and Setting

We conducted a nested case–control study with prospective enrollment of a cohort of individuals undergoing evaluation for possible TB disease in 2 public primary care centers serving the Central and Western Districts of Cali, Colombia, between 31 July 2023 and 30 August 2024. These facilities function as primary diagnostic centers for their catchment populations and offer TB diagnostic evaluation and treatment services free of charge. Healthcare staff are encouraged to follow clinical practice guidelines from WHO and the Colombia Ministry of Health recommending microbiologic testing for those with TB symptoms (ie, cough, fever, chills, night sweats, weight loss) lasting ≥2 weeks [14]. In addition, testing is recommended regardless of TB symptom duration for priority groups, including close contacts of persons with TB, the immunosuppressed, and individuals with a chest radiograph suggestive of TB [12, 14].

### Study Population

A trained study physician and nurse assistant shadowed routine healthcare staff at study facilities as they evaluated inpatients and outpatients for TB disease. We invited individuals aged ≥7 years whom clinicians ordered microbiologic evaluation for TB to participate in the study. To capture all eligible individuals, study staff reviewed laboratory records and invited consecutive nonreferred individuals meeting inclusion criteria to participate. They excluded individuals currently receiving TB treatment, those reporting prior TB treatment or TB preventive therapy within the preceding 12 months, and persons who were incarcerated.

### Study Procedures

Research staff collected participant demographics and clinical information using electronic case record forms (REDCap) [15]. Healthcare personnel instructed participants to expectorate 3 good-quality sputum samples, with at least 1 early morning specimen and 2 on the spot, each into a sterile, wide-mouth, spill-proof container [16]. They referred those unable to expectorate to on-site respiratory therapists for sputum induction (Supplementary methods) [17]. Samples were examined by smear microscopy using acid-fast staining, with 1 sent for liquid culture in mycobacterial growth indicator tubes (MGITs) either at on-site health center laboratories or at a third-party laboratory as part of routine diagnostic procedures. Local providers typically perform a single sputum mycobacterial culture; we therefore adopted this as a pragmatic reference standard. We defined pulmonary TB as present in anyone with ≥1 baseline sputum MGIT culture positive for *Mycobacterium tuberculosis* (*Mtb*) complex. We evaluated the discomfort and acceptability of saliva and swab sample collection as detailed in the Supplementary methods.

For research-related molecular testing, research staff collected saliva and oral swabs (hereafter referred to as swabs) from participants on the same day, after instructing them to cough lightly up to 5 times to mobilize posterior oropharyngeal material. Up to 4 oral samples were collected using sterile flocked nylon swabs (Copan FLOQSwabs®, Murrieta, CA) by simultaneously sweeping the buccal surfaces with 2 swabs in the following sequence: cheeks, sublingual area, soft palate, and tongue. We prioritized a multi-surface sweep to ensure comprehensive sampling and used nylon-flocked swabs to maximize capture and release of surface *Mtb* DNA, as previously described [18]. After collection, swabs were placed in dry, sterile containers. For saliva collection, participants were instructed to follow the same coughing procedures as before swab collection and then to deposit 1–5 mL of saliva into a sterile, wide-mouth, spill-proof container by drooling [19]. Saliva and swabs were collected ≥30 minutes after the last food or beverage intake and before sputum collection whenever possible. All samples were refrigerated (2–8°C) for ≤72 hours prior to transport and storage at −80°C in our research biorepository.

### Sample Processing and Molecular Testing

Within the prospective cohort, we matched persons with culture-confirmed pulmonary TB to controls without microbiological evidence of pulmonary TB using 1:1 incidence-density sampling by collection date, site, age (<18, 18–59, and ≥60 years), and sex (Supplementary methods). We excluded participants without culture results. In a BSL-2 cabinet, a microbiologist blinded to participant characteristics and reference standard results eluted thawed swabs in a 1:1 solution of 1× DPBS (Dulbecco's Phosphate-Buffered Saline, Gibco, Thermo Fisher, Waltham, MA) and sample reagent (SR, Cepheid, Sunnyvale, CA) in a 15 mL conical tube to a final volume of 3 mL. We mixed thawed saliva with an equal volume of SR (1.5 mL each). After vortexing for 10 minutes, 2 mL of each preparation was loaded into Xpert-Ultra cartridges (Cepheid) for testing following the manufacturer's instructions. We repeated any assay returning Invalid, Error, or No Result.

### DNA Biomass in Saliva and Oral Swab Samples

As a surrogate for *Mtb* DNA recovery from the oral cavity, we quantified human DNA in paired saliva and swab specimens from a subset of participants. Cell counts were estimated from a U-937 standard DNA curve (Supplementary methods). We also compared the median lowest Xpert-Ultra cycle threshold (Ct) values by sample type as a measure of *Mtb* biomass recovery.

### Sample Size Calculation

Assuming 85% sensitivity for molecular testing on oral samples, we estimated that 100 persons with microbiologically confirmed TB would allow estimation of sensitivity with ~±7% precision using exact binomial 95% confidence intervals (CIs); assuming a 10% culture positivity, we sought to enroll 1000 people with possible TB. Assuming 98% specificity, 74 non-TB participants would yield ±3.2% precision using exact binomial 95% CI [20], but given the matched design, we enrolled equal numbers of controls and cases.

### Statistical Analysis

We summarized categorical variables as proportions and continuous variables as medians (IQR, interquartile range). We estimated Xpert-Ultra sensitivity and specificity independently for saliva and swabs using sputum MGIT culture as the reference standard and classifying trace or higher results as *Mtb* positive. We compared the diagnostic accuracy of saliva versus swab using McNemar's test [21] with mid-*P* correction [22]. To compare diagnostic likelihood ratios between saliva and swab, we estimated the bootstrap distribution of each likelihood ratio (positive and negative) independently for each medium. If the 95% range of the distribution excluded the null value (1), we inferred a *P*-value <.05. Subgroup heterogeneity was evaluated with a 2-sided Fisher's exact test with *P* < .0031 after Bonferroni correction. Analyses were conducted in Stata 16.0 (StataCorp, College Station, TX), figures were generated in R 4.2.3 [23], and reporting followed the STARD guidelines [24] (Supplementary Supplement 1).

### Human Subjects' Protection

Institutional review boards at CIDEIM (#1325), Yale University (#2000034128), and the Cali Secretary of Public Health (#202341450100044831) approved the study protocol. All participants provided written informed consent, and minors provided written assent.

## RESULTS

### Clinical and Demographic Characteristics

**Figure 1.**
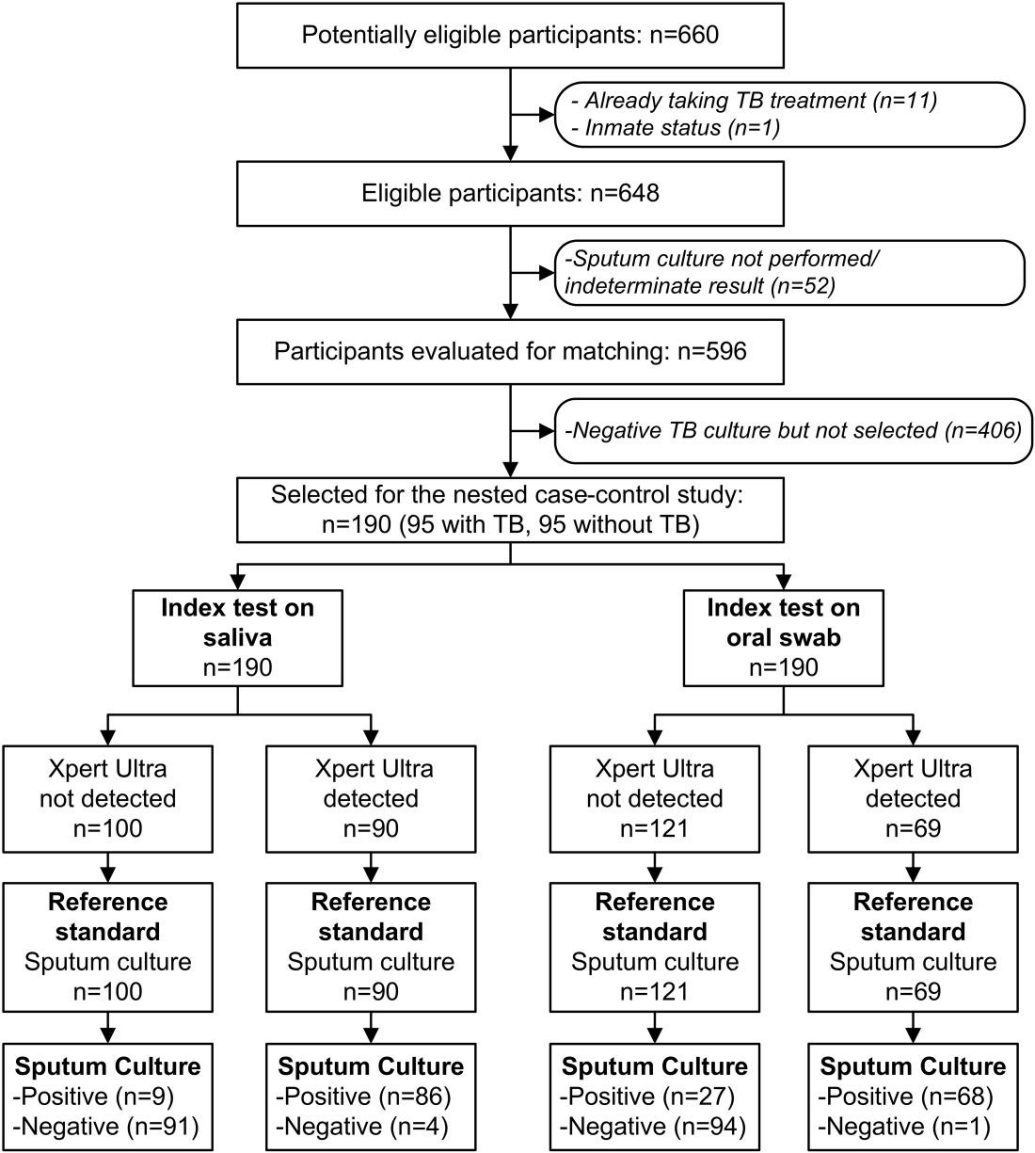
STARD diagram illustrating the flow of participants in the nested case–control study. Three saliva assays and 1 swab assay had to be repeated because the initial Xpert-Ultra gave the result “Invalid.” Abbreviation: TB, tuberculosis.

We screened a total of 660 individuals, excluding 12 who did not meet eligibility criteria. All 648 enrolled participants (Figure 1) provided saliva, swab, and sputum samples. Final culture results were available for 596 participants, excluding 52 individuals for whom sputum culture was not performed or the results were indeterminate. Ninety-five (15.9%) had culture-confirmed TB.

After matching, there were 190 participants with paired swabs and saliva available for index testing. Baseline characteristics (Table 1) showed a median age of 42.5 years (IQR: 29–57 years) and a predominance of men (78%). Most participants reported Mestizo ethnicity (n = 123, 65%). Fifty-nine percent (n = 113) were recruited from outpatient settings.

**Table 1.** Baseline Demographic and Clinical Characteristics of Participants in the Diagnostic Accuracy Analysis

Characteristics	TB	Not TB	All Participants
n (%)^a^	(n = 95)	(n = 95)	(n = 190)
Age, yr, median (IQR)	41 (27–56)	44 (32–58)	42.5 (29–57)
Male sex	74 (77.9)	74 (77.9)	148 (77.9)
Ethnicity			
Mestizo	66 (69.5)	57 (60.0)	123 (64.7)
Black/Afro-Colombian	22 (23.1)	31 (32.6)	53 (27.9)
Other^b^	7 (7.4)	7 (7.4)	14 (7.4)
Healthcare setting			
Outpatient	51 (53.7)	62 (65.3)	113 (59.5)
Inpatient	44 (46.3)	33 (34.7)	77 (40.5)
Pre-existing conditions			
HIV (1 or 2)	8 (8.4)	12 (12.6)	20 (10.5)
Asthma/COPD	4 (4.2)	12 (12.6)	16 (8.4)
Diabetes mellitus	4 (4.2)	3 (3.1)	7 (3.7)
History of TB	6 (6.3)	7 (7.4)	13 (6.8)
Symptoms			
Cough ≥2 wk duration	86 (90.5)	88 (92.6)	174 (91.6)
Fever	69 (72.6)	54 (56.8)	123 (64.7)
Weight loss	85 (89.5)	59 (62.1)	144 (75.8)
Night sweats	71 (74.7)	55 (57.9)	126 (66.3)
Swab/saliva collected after sputum expectoration	42 (44.2)	39 (41.1)	81 (42.6)
Sputum bacillary load			
Sputum smear microscopy positive^c^	69 (74.2)	1 (1.3)	70 (40.5)
Time to culture positivity, median (IQR), d	12 (8–16)	…	12 (8–16)

Abbreviations: COPD, chronic obstructive pulmonary disease; HIV, human immunodeficiency virus; IQR, interquartile range; TB, tuberculosis.

^a^Unless otherwise specified.

^b^Other includes White and Indigenous.

^c^Smear microscopy results were missing for 17 participants, including 2 with culture-confirmed TB and 15 who did not have TB.

Eleven percent of participants were PWH (n = 20), and 7% had previously been treated for TB (n = 13). The most frequent symptom was cough of ≥2 weeks duration (n = 174, 92%). Only 13% (n = 24) required sputum induction by respiratory therapy. Sputum smear microscopy was positive in 69 of 93 individuals with culture-confirmed TB who had smear results available (74.2%; Table 1).

Index tests were performed at a median of 119 days after sample collection (IQR: 81.5–154.6). We repeated 4 assays, including 3 saliva samples and 1 swab, because the initial Xpert-Ultra gave the result “Invalid.”

### Diagnostic Accuracy of Xpert-Ultra on Saliva and Oral Swabs

Saliva achieved 90.5% sensitivity (95% CI, 82.8–95.6) and 95.8% specificity (95% CI, 89.6–98.8), whereas swabs yielded 71.6% sensitivity (95% CI, 61.4–80.4) and 98.9% specificity (95% CI, 94.3–100). Saliva was therefore more sensitive than swabs by an absolute difference of 18.9% (95% CI, +10.0 to +27.9; *P* < .001). There was not a statistically significant difference in specificity between saliva and swabs (−3.2%, 95% CI, −7.7 to +1.4, *P* = .25; Table 2).

**Table 2.** Diagnostic Performance of Oral Swabs and Saliva With Xpert-Ultra Testing for Detecting Pulmonary Tuberculosis, Using Sputum Liquid Culture as the Reference Standard

Parameter	Saliva	Oral Swab	*P*-Value
	(n = 190)	(n = 190)	
Counts, n			
True positive	86	68	…
False positive	4	1	…
False negative	9	27	…
True negative	91	94	…
Diagnostic accuracy, % (95% CI)			
Sensitivity	90.5	71.6	<.001^a^
	(82.8–95.6)	(61.4–80.4)	
Specificity	95.8	98.9	.25^a^
	(89.6–98.8)	(94.3–100)	
Likelihood ratios, (95% CI)			
LR+	21.5	68.0	≥.05^b^
	(8.2–56.2)	(9.6–479.7)	
LR–	0.10	0.29	<.05^b^
	(0.05–0.18)	(0.21–0.40)	

Abbreviations: CI, confidence interval; LR, likelihood ratio.

^a^McNemar's test with mid-*P* correction.

^b^Estimated from the bootstrap distribution.

**Figure 2.**
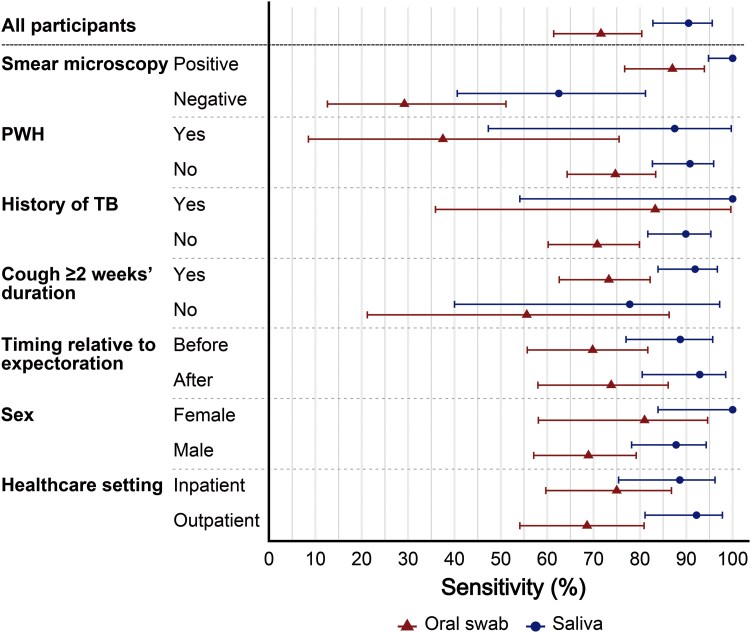
Sensitivity of saliva and oral swab samples for the diagnosis of pulmonary tuberculosis, by subgroup. Forest plot showing sensitivity with 95% confidence intervals for each sample type, oral swab and saliva, across different participant subgroups, including: smear microscopy status, HIV status, history of TB, cough duration, timing relative to expectoration (before or after expectoration), sex and healthcare setting (inpatient vs outpatient). Abbreviations: PWH, people with HIV; TB, tuberculosis.

Saliva consistently outperformed swabs across all subgroups (Figure 2) including those requiring sputum induction, for whom we observed higher sensitivity for saliva (75%) than for swab (62.5%) (Supplementary Table 1). Sensitivity of saliva was 62.5% among smear-negative individuals versus 100% among smear-positive individuals (*P* < .001). For swabs, sensitivity was only 29.2% among smear-negative persons but 87% among smear-positive persons (*P* < .001). Specificity of swabs ranged from 96.8% to 100%, and that of saliva from 83.3% to 100% (Supplementary Figure 1). Sensitivity and specificity of both saliva and swab were modestly higher for samples collected after expectoration compared to those collected before expectoration, although the differences did not reach statistical significance. Saliva sensitivity was 88.7% before versus 92.9% after expectoration (*P* = .73), and specificity was 94.6% before versus 97.4% after expectoration (*P* = .64). Similarly, swab sensitivity was 69.8% before and 73.8% after (*P* = .82), while specificity was 98.2% before and 100% after expectoration (*P* = 1.0).

Four male participants, aged 19–28 years, were *Mtb*-positive on saliva but sputum culture-negative; one was also positive on the paired swab. The first, a 19 year old, had very low levels of *Mtb* DNA detected in both saliva and swab; culture was negative, and smear was not available. The second, a 21 year old with HIV and malnutrition, had a small amount of DNA amplified in saliva only, while swab, smear, and culture were all negative. Because of persistent symptoms, both individuals underwent repeat cultures that subsequently grew *Mtb*, confirming their positive index test results as true positives. The third participant, a 26 year old with a history of TB treated 12 months earlier, yielded a trace-positive saliva Xpert-Ultra signal while all other tests were negative, most consistent with a false positive result. The fourth participant, a 28 year old, had a low-intensity *Mtb* DNA signal in saliva, with swab, sputum smear, and culture remaining negative; his symptoms resolved without treatment, suggesting a false positive saliva result, paucibacillary TB disease that reverted to subclinical TB, or active TB with spontaneous cure.

### Biomass Quantification and Semi-quantitative Xpert-Ultra Results Between Saliva and Swabs

**Figure 3.**
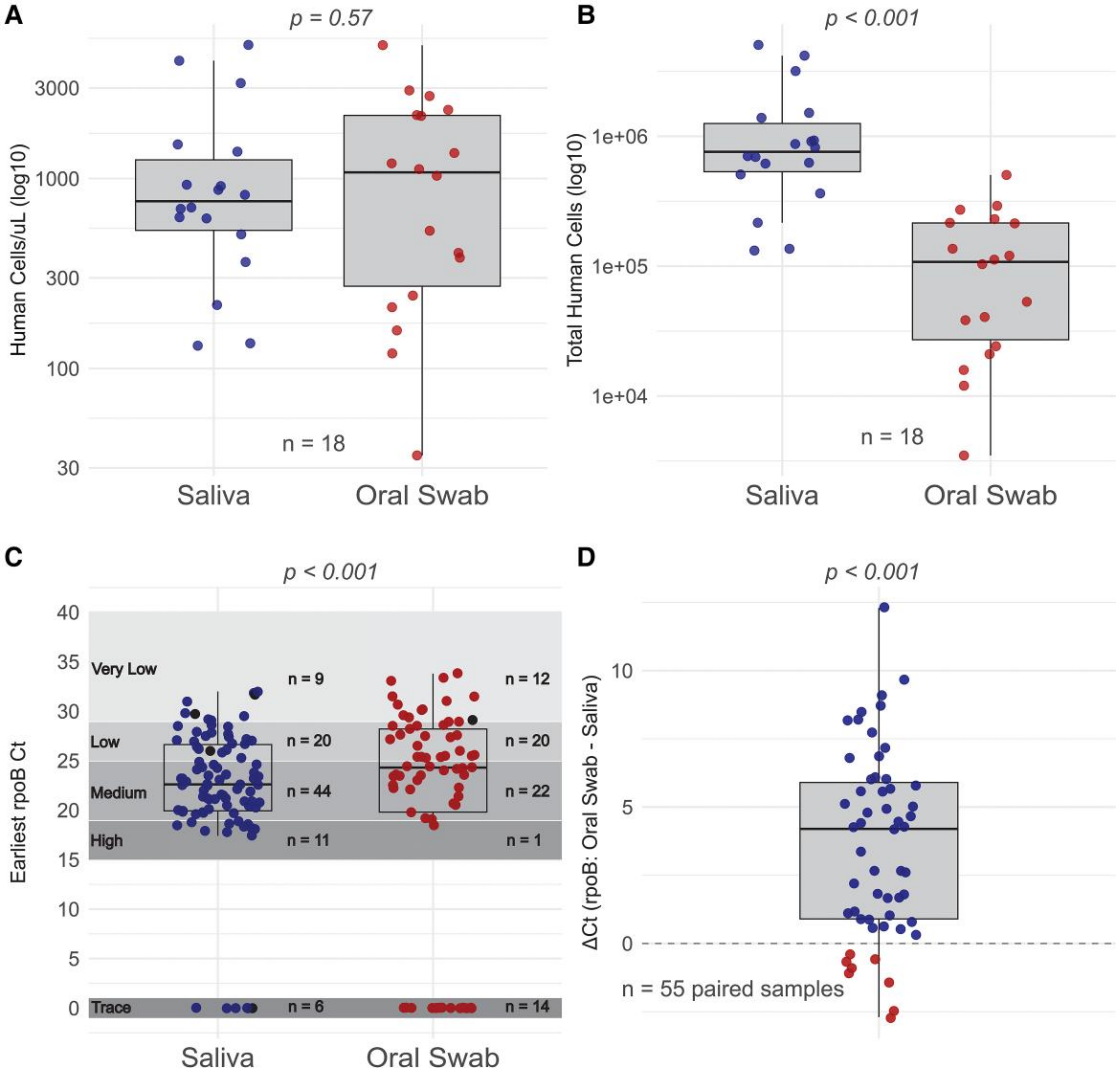
Quantification of human and *Mycobacterium tuberculosis* (*Mtb)* cellular biomass in saliva and oral swabs. The top 2 panels display the human cell biomass quantification by RNAseP among 18 *Mtb-*culture-negative participants. (*A*) shows the concentration of cells in saliva and in swab eluate, as assessed by real-time PCR for human RNAseP (*P* = .57 for difference in number of cells by the Wilcoxon signed-rank test). (*B*) shows the estimated number of cells input into the Xpert-Ultra assay for saliva at the standard 1 mL input volume and for oral swab at the standard 0.1 mL input volume (*P* < .001 for difference in number of cells by the Wilcoxon signed-rank test). (*C*) presents the quantitative (cycle threshold [Ct] values for the earliest *rpo*B amplicon detected) and semiquantitative results based on those Ct values (trace, high, medium, low, and very low) for those with positive Xpert index tests on saliva (n = 90) or on oral swab (n = 69) (*P* < .001 when compared with sample types using the Mann–Whitney *U* test). (*D*) presents the differences in Ct values for the 55 participants positive on both saliva and swabs, after excluding trace positive results for which Ct values are not reported (Wilcoxon signed-rank test, *P* < .001).

We quantified human biomass in paired saliva and swab samples from 18 culture-negative participants enrolled February–March 2024. Cell counts per microliter did not differ statistically significantly between sample types, although swab values were more widely dispersed (Figure 3*A*). After accounting for the Xpert-Ultra loading volumes—1 mL for saliva versus ∼80–100 µL for a swab—total cell numbers were higher in saliva (*P* < .001; Figure 3*B*). Among Ultra-positive pairs (ie, true and false positives) in the nested case–control set, we plotted the lowest *rpoB* cycle threshold (Ct) value for each specimen (*P* < .001; Figure 3*C*). Saliva yielded lower Ct values than swabs (*P* < .001; Figure 3*D*).

### Discomfort and Acceptability of Sample Collection Methods

Among 171 participants (median age 53 years, IQR, 33–66), 76% (67/88) in the saliva group and 82% (68/83) in the swab group reported no discomfort with sample collection. Both methods were highly acceptable (>95% rated them favorably), with no differences in discomfort (*P* = .25) or acceptability (*P* = .77) by sample type (Supplementary results).

## DISCUSSION

In this prospectively designed, nested case–control study, we evaluated the diagnostic performance of Xpert-Ultra on saliva and swabs for TB detection. Saliva testing exceeded the WHO sensitivity target for a low-complexity, nonsputum test (≥80%), whereas swabs did not. Both specimen types achieved high specificity: swabs met the WHO target of ≥98%, and while saliva was slightly less specific, the difference was not statistically significant [5, 6]. After reclassifying 2 of 4 saliva-positive, culture-negative cases and the 1 swab-positive, culture-negative case as true positive TB diagnoses based on follow-up clinical and microbiologic data, specificity was 98% for saliva and 100% for swabs.

The sensitivity of both saliva and swabs paired with Xpert-Ultra exceeded 80% among smear-positive individuals, those with a history of TB, and women. Saliva also exceeded 80% in most other strata, except for smear-negative persons, those with <2 weeks of cough, and individuals requiring sputum induction. However, positive smear status was the only statistically significant determinant of higher performance. Despite our use of a previously standardized oral-swab collection protocol involving comprehensive sampling of the oral cavity, our paired analyses showed that a single swab recovered a similar concentration of human biomass as saliva. However, because the loaded volume was ∼10-fold lower for swabs (∼0.1 mL) than for saliva (1 mL), the total biomass input was statistically significantly lower for swabs. To the extent that recovery of human and *Mtb* biomass from the oral cavity is correlated, lower input volumes may explain the lower sensitivity of swabs compared to saliva.

Prior studies have shown that saliva-based molecular tests for TB have high specificity but variable sensitivity (ranging 38%–90%) [9, 10, 25, 26]. The proportions of symptomatic participants and of smear-positive participants in our study were similar to a previous study from Uganda, which similarly reported excellent sensitivity of 90% for saliva Xpert-Ultra against a composite reference standard of 2 sputum cultures, as well as a lower sensitivity among smear-negative than among smear-positive persons [9]. A multicenter study from China reported a slightly lower saliva sensitivity of only 79% among a cohort of fewer symptomatic (70%) and mostly smear-negative participants using posterior oropharyngeal saliva [10]. The lower performance in the latter study may reflect case-mix differences associated with a more paucibacillary spectrum of disease [27].

For swabs, studies report sensitivities ranging from 22% to 92% with near-perfect specificity, with higher sensitivity in studies conducted in settings with fewer paucibacillary cases and those employing tongue sampling or more intensive pre-analytic processing [7, 28–36]. A study from Uganda performing Xpert-Ultra on 2 simultaneously collected and assayed Copan FLOQSwabs yielded a sensitivity of 72%, similar to our study, although in reference to multiple sputum cultures [35]. A recent preprint examined the performance of tongue swabs across diverse regions (Southeast Asia, Africa, and South America; n = 1844) and reported 66% sensitivity (site range 37%–78%) and 99% specificity versus sputum cultures among a paucibacillary population (12.5% smear-positive); among PWH, sensitivity was 50% [28].

A principal advantage of noninvasive sample collection methods like swab and saliva is that they can be self-administered with minimal guidance, whether at home, during community outreach, or in clinics, making these samples especially attractive for anyone who may have more difficulty expectorating sputum, including children, PWH, persons with nonproductive cough, and those without symptoms [37–39]. In our study, for example, only a quarter of participants reported any discomfort, and nearly all judged the collection method acceptable. Because our evaluation involved symptomatic individuals in a clinical setting, performance may be overestimated for populations in whom non-sputum testing would be most useful. Therefore, additional studies are needed in community or home-based settings—where bacillary loads may be lower—including among asymptomatic individuals and children [29, 40]. Both saliva and swabs could potentially reduce reliance on more invasive procedures such as induced sputum, gastric aspiration, and bronchoalveolar lavage, while expanding access to molecular testing during active case-finding and systematic screening activities [40].

Our study had several strengths. First, the nested case–control design enhances efficiency while minimizing selection bias. Second, our collection of paired saliva and swabs in the same participants enabled head-to-head evaluation of nonsputum specimens. Third, we collected and reported a variety of secondary measures, including implementation outcomes and measures of DNA recovery from the oral cavity and from different specimen types, to contextualize the observed differences in performance.

Our study also had limitations. First, relying on a single sputum mycobacterial culture as the reference standard may have missed paucibacillary TB, biasing sensitivity upward and specificity downward compared to studies using multiple sputum cultures or a composite reference standard [25]. Second, we also lacked a sputum molecular testing comparator. Third, only 27% of participants were smear negative and only 3% were aged <18 years, limiting generalizability to these groups [10]. Fourth, we instructed participants to cough lightly before sample collection, which might enhance swab and saliva sensitivity but at the cost of greater infection control risk. Finally, nearly half of index tests were collected after sputum expectoration, a potential source of inflated saliva and swab sensitivity, although we did not observe statistically significant differences in sensitivity by the timing of sample collection.

In summary, saliva is a potentially robust nonsputum option for patients unable to produce sputum. Swabs provide a practical alternative when saliva or sputum is unavailable, although with lower sensitivity. Future work should optimize biomass recovery, processing, and concentration to increase sensitivity without putting laboratory staff at risk of exposure to *Mtb* aerosols during testing. In parallel, studies should assess the feasibility and accuracy of saliva and swab-based molecular testing for passive case finding in paucibacillary individuals—including children and those who cannot expectorate sputum—as well as for active case finding among those with subclinical TB.

## Supplementary Material

ciag055_Supplementary_Data

## Supplementary Data

Supplementary materials are available at *Clinical Infectious Diseases* online. Consisting of data provided by the authors to benefit the reader, the posted materials are not copyedited and are the sole responsibility of the authors, so questions or comments should be addressed to the corresponding author.
