# Supplementary material for: Diagnostic Accuracy of Molecular Testing on Saliva and Oral Swabs for Pulmonary Tuberculosis
Source: Clin Infect Dis. 2026 Mar 12;82(6):e1313–21. doi: 10.1093/cid/ciag055 (PMC13341261; doi:10.1093/cid/ciag055)
Supplement: ciag055_Supplementary_Data [file ciag055_supplementary_data.zip › Supplementary methods.pdf]

Supplementary methods

- **Methods Used to Evaluate Discomfort and Acceptability** 2
- **Human DNA biomass in saliva and oral swab samples** 4
  - **Table S3.** 4
- **Sputum induction** 6
- **Matching Approach** 7
  - **Table S4.** 7

Methods Used to Evaluate Discomfort and Acceptability

Study Design and Participants

We conducted a nested cross-sectional analysis. A consecutive subsample of participants was invited to complete a questionnaire designed to jointly assess discomfort and acceptability of saliva or oral swab collection methods. A trained researcher administered the questionnaire to consent participants immediately after the completion of sample collection (within 5 to 10 minutes). Each participant was evaluated for only one of the methods, either saliva or oral swab, even if both types of samples were provided. Records of participants who did not complete all questionnaire items were excluded.

Questionnaire and Validation

Discomfort was measured using a single-item, self-reported 0–10 numeric rating scale (NRS), where 0 represented no discomfort and 10 the worst imaginable discomfort. The scale was adapted from the standard NRS [1] to assess participants' subjective discomfort related to sample collection. Prior to administration of the acceptability questionnaire, we translated and culturally adapted the Acceptability of Intervention Measure (AIM) [2] for use in Colombia. Between December 2023 and January 2024, we conducted cognitive interviews to confirm participants' understanding of AIM, resulting in three interrelated items that assess the same acceptability construct: 1) "The sample collection seems acceptable to me," 2) "I think the sample collection is good," and 3) "I think the sample collection is tolerable,". Each statement was rated on a 5-point Likert scale ranging from strongly disagree to strongly agree.

Data Management and Statistical Analysis

All analyses were conducted using Stata version 17 (StataCorp, College Station, TX). Descriptive statistics were used to summarize demographic characteristics and outcome variables. Age, discomfort score, and acceptability score were reported using median and interquartile range (IQR). Categorical variables (e.g., sex, sample type and individual acceptability-item responses) were summarized using frequencies and percentages. We assessed normality of continuous variables using the Shapiro–Wilk test.

We compared discomfort score by sample types and between sex using the Mann–Whitney U test (Wilcoxon rank-sum for independent samples), and we examined the association between discomfort and age using Spearman's rank correlation.

For acceptability, we calculated Cronbach's alpha across the three items to confirm internal consistency and justify aggregation [3,4], and then generated a continuous acceptability score by averaging the three item responses. We then assessed its distribution with the Shapiro–Wilk test in the full sample as well as stratified by sample type and sex. We compared the median acceptability score between sample types and between sexes using the Mann–Whitney U test and examined its correlation with age and discomfort score via Spearman's rank correlation. Finally, we applied median (τ=0.5) quantile regression [5] to model the acceptability score as a function of age, discomfort score, sample type, and sex, estimating each predictor's effect on the conditional median.

References

1. Hjermstad MJ, Fayers PM, Haugen DF, et al. Studies comparing numerical rating scales, verbal rating scales, and visual analogue scales for assessment of pain intensity in adults: A systematic literature review. J Pain Symptom Manage **2011**; 41:1073–1093.
2. Weiner BJ, Lewis CC, Stanick C, et al. Psychometric assessment of three newly developed implementation outcome measures. Implementation Science **2017**; 12:108. Available at: https://doi.org/10.1186/s13012-017-0635-3.

3. Cronbach LJ. Coefficient alpha and the internal structure of tests. Psychometrika **1951**; 16:297–334. Available at: https://doi.org/10.1007/BF02310555.
4. Tavakol M, Dennick R. Making sense of Cronbach's alpha. Int J Med Educ **2011**; 2:53–55. Available at: http://www.ijme.net/archive/2/cronbachs-alpha/.
5. Koenker R. Quantile Regression. Cambridge: Cambridge University Press, **2005**. Available at: https://www.cambridge.org/core/product/C18AE7BCF3EC43C16937390D44A328B1.

Human DNA biomass in saliva and oral swab samples

Human DNA was quantified in paired saliva and oral swab specimens from a subset of 18 participants as a proxy for sample biomass.

Standard curve generation

A seven-point standard curve (10^6 to 10^0 cell equivalents) was prepared from U-937 cells (3×10^6 input). DNA was extracted with the DNeasy® Blood & Tissue Kit (Qiagen, Hilden, Germany) and eluted in 30 µL AE buffer. A 10 µL aliquot corresponding to 10^6 cell equivalents (STD-1) was serially diluted 1:10 to generate STD-2 to STD-7 (10^5 to 10^0).

qPCR targeted the human RNase P gene using TaqMan chemistry (forward 5′-AGATTTGGACCTGCGAGCG-3′; reverse 5′-GAGCGGCTGTCTCCACAAGT-3′; probe 5′-(Cy5)TTCTGACCTGAAGGCTCTGCGCG(BHQ3)-3′). Reactions (25 µL) contained 10 µL DNA, 10 µL 2× TaqMan™ Gene Expression Master Mix (Applied Biosystems), 1 µL of each 10 µM primer, 0.5 µL 5 µM probe, and 2.5 µL water. Cycling on a CFX96 (Bio-Rad): 95 °C 5 min; 45 cycles of 95 °C 15 s and 60 °C 60 s.

Standard-curve performance: Ct (10^6–10^0) = 22.18, 25.06, 28.78, 32.52, 36.31, 38.38, 43.08; slope −3.480, intercept 42.818, R^2 0.994, efficiency 93.8%.

Specimen processing

Swabs were thawed and eluted in 1× DPBS (Gibco) and Xpert sample reagent (Cepheid) 1:1 to a final volume of 300 µL. Saliva was mixed 1:1 with sample reagent (150 µL + 150 µL; final 300 µL). All tubes were vortexed 1 min, incubated at ~23 °C for 10 min, vortexed 1 min, and rested 5 min at room temperature.

DNA extraction and qPCR

DNA was extracted from the entire 300 µL volume (DNeasy® Blood & Tissue Kit) and eluted in 30 µL. RNase P qPCR was performed as above.

Quantification

Cell equivalents per reaction were derived from the standard curve as: cells = $10^{((Ct - intercept)/slope)}$. Values were converted to cells/µL in the eluate and total cells per specimen using assay volumes.

Per-sample Ct values

Individual Ct values for saliva (S) and swab (H) specimens are provided in Supplementary Table 3.

Table S3.

#	Ct S	Ct H
TB-02-0106	27.00	24.31
TB-02-0091	26.02	23.96
TB-02-0083	26.18	28.67
TB-02-0076	25.61	24.32
TB-02-0049	24.97	25.00

TB-02-0042	27.79	27.61
TB-02-0037	26.00	25.19
TB-02-0032	23.02	26.83
TB-01-0252	25.68	23.85
TB-01-0241	26.49	25.29
TB-01-0239	23.31	23.03
TB-01-0238	23.72	24.21
TB-01-0231	26.20	25.42
TB-01-0210	28.53	30.54
TB-01-0195	25.77	26.92
TB-01-0187	25.58	27.83
TB-01-0180	28.48	26.42
TB-01-0174	24.84	28.25

Sputum Induction

Sputum induction was performed by respiratory therapists under airborne precautions. Participants first performed an alcohol-free mouth rinse, were pre-medicated with inhaled salbutamol (200 mg via spacer), and observed for 15 minutes. Nebulized hypertonic saline (3–5%; 5 CC, room temperature) was then delivered for ~10 minutes at 6–8 L/min. Once a productive cough was elicited, sputum was collected into a sterile container. For these patients, index-test specimens (saliva and swabs) were collected 30 minutes after completion of the sputum-induction procedure.

Matching Approach

We applied incidence-density (risk-set) matching to control for temporal, site-level, and demographic variability. For each culture-confirmed case, one control with a negative sputum culture was selected from the concurrent risk set, matched 1:1 on: (i) sample-collection date (nearest available), (ii) study site, (iii) age group (<18, 18–59, ≥60 years), and (iv) sex. Controls met the same eligibility criteria as cases and provided both saliva and an oral swab at baseline.

When multiple eligible controls were available, we selected the individual with the smallest calendar-date difference; ties were resolved deterministically by dataset order, and the chosen control was removed from the pool to prevent reuse. Matching yielded 95 pairs (n = 190).

The procedure was implemented via an R script that enforces these criteria; Annex 2 provides the matching output and audit trail. A 1:1 ratio was prespecified based on power considerations for the primary endpoint (sensitivity, driven by the number of cases) and operational constraints, and to facilitate paired analyses.

Table S4.

Supplementary Table 4. Matching pairs for the accuracy analysis.

reccord_id	pair_id	cult_result	study_site_matched	age	gender_matched	days_diff
TB-01-0001	1	0	yes	69	yes	0
TB-01-0002	1	1	yes	61	yes	
TB-01-0021	2	1	yes	63	yes	1
TB-01-0030	2	0	yes	79	yes	
TB-01-0034	3	1	yes	24	yes	0
TB-01-0036	3	0	yes	35	yes	
TB-01-0044	4	1	yes	38	yes	0
TB-01-0045	4	0	yes	55	yes	
TB-01-0083	5	1	yes	24	yes	2
TB-01-0085	5	0	yes	45	yes	
TB-01-0108	6	0	yes	25	yes	7
TB-01-0111	6	1	yes	48	yes	
TB-01-0112	7	1	yes	59	yes	6
TB-01-0119	7	0	yes	55	yes	
TB-01-0100	8	0	yes	28	yes	33
TB-01-0114	8	1	yes	59	yes	
TB-01-0113	9	0	yes	64	yes	3
TB-01-0115	9	1	yes	68	yes	

TB-01-0098	10	0	yes	40	yes	43
TB-01-0120	10	1	yes	36	yes	
TB-01-0159	11	1	yes	53	yes	4
TB-01-0167	11	0	yes	20	yes	
TB-01-0168	12	1	yes	29	yes	1
TB-01-0169	12	0	yes	26	yes	
TB-01-0184	13	1	yes	22	yes	1
TB-01-0186	13	0	yes	59	yes	
TB-01-0209	14	0	yes	31	yes	0
TB-01-0211	14	1	yes	40	yes	
TB-01-0214	15	1	yes	44	yes	1
TB-01-0216	15	0	yes	34	yes	
TB-01-0223	16	0	yes	41	yes	3
TB-01-0232	16	1	yes	22	yes	
TB-01-0224	17	0	yes	43	yes	7
TB-01-0237	17	1	yes	33	yes	
TB-01-0246	18	1	yes	22	yes	8
TB-01-0257	18	0	yes	52	yes	
TB-01-0259	19	1	yes	56	yes	24
TB-01-0269	19	0	yes	56	yes	
TB-01-0260	20	1	yes	35	yes	20
TB-01-0270	20	0	yes	26	yes	
TB-01-0265	21	1	yes	31	yes	7
TB-01-0273	21	0	yes	33	yes	
TB-01-0267	22	1	yes	59	yes	33
TB-01-0284	22	0	yes	48	yes	
TB-01-0274	23	1	yes	54	yes	30
TB-01-0286	23	0	yes	44	yes	
TB-01-0277	24	1	yes	30	yes	28
TB-01-0288	24	0	yes	25	yes	
TB-01-0280	25	1	yes	41	yes	20
TB-01-0295	25	0	yes	44	yes	

TB-01-0283	26	1	yes	46	yes	17
TB-01-0298	26	0	yes	35	yes	
TB-01-0285	27	1	yes	29	yes	19
TB-01-0299	27	0	yes	48	yes	
TB-01-0290	28	1	yes	29	yes	0
TB-01-0291	28	0	yes	32	yes	
TB-01-0292	29	1	yes	34	yes	22
TB-01-0306	29	0	yes	28	yes	
TB-01-0293	30	0	yes	59	yes	7
TB-01-0296	30	1	yes	34	yes	
TB-01-0302	31	1	yes	23	yes	0
TB-01-0303	31	0	yes	35	yes	
TB-01-0304	32	1	yes	70	yes	2
TB-01-0314	32	0	yes	70	yes	
TB-01-0305	33	1	yes	39	yes	2
TB-01-0311	33	0	yes	23	yes	
TB-01-0307	34	1	yes	25	yes	1
TB-01-0310	34	0	yes	44	yes	
TB-01-0308	35	1	yes	42	yes	1
TB-01-0312	35	0	yes	44	yes	
TB-01-0313	36	1	yes	81	yes	0
TB-01-0318	36	0	yes	63	yes	
TB-01-0316	37	1	yes	38	yes	0
TB-01-0317	37	0	yes	32	yes	
TB-01-0322	38	1	yes	25	yes	2
TB-01-0324	38	0	yes	23	yes	
TB-01-0327	39	1	yes	81	yes	2
TB-01-0331	39	0	yes	75	yes	
TB-01-0345	40	1	yes	69	yes	8
TB-01-0351	40	0	yes	65	yes	
TB-01-0340	41	0	yes	26	yes	6
TB-01-0346	41	1	yes	41	yes	

TB-01-0349	42	1	yes	41	yes	1
TB-01-0350	42	0	yes	55	yes	
TB-01-0347	43	0	yes	70	yes	6
TB-01-0352	43	1	yes	74	yes	
TB-01-0353	44	0	yes	24	yes	1
TB-01-0354	44	1	yes	20	yes	
TB-01-0355	45	1	yes	58	yes	7
TB-01-0363	45	0	yes	35	yes	
TB-01-0356	46	1	yes	60	yes	12
TB-01-0366	46	0	yes	21	yes	
TB-01-0359	47	0	yes	71	yes	1
TB-01-0362	47	1	yes	72	yes	
TB-01-0364	48	1	yes	46	yes	14
TB-01-0372	48	0	yes	59	yes	
TB-01-0368	49	1	yes	55	yes	8
TB-01-0374	49	0	yes	53	yes	
TB-01-0376	50	0	yes	88	yes	5
TB-01-0379	50	1	yes	73	yes	
TB-02-0012	51	1	yes	38	yes	5
TB-02-0016	51	0	yes	56	yes	
TB-02-0011	52	0	yes	45	yes	19
TB-02-0015	52	1	yes	54	yes	
TB-02-0019	53	1	yes	42	yes	0
TB-02-0020	53	0	yes	31	yes	
TB-02-0026	54	0	yes	43	yes	0
TB-02-0027	54	1	yes	45	yes	
TB-02-0025	55	0	yes	36	yes	10
TB-02-0030	55	1	yes	45	yes	
TB-02-0031	56	1	yes	26	yes	12
TB-02-0041	56	0	yes	34	yes	
TB-02-0035	57	1	yes	47	yes	11
TB-02-0045	57	0	yes	38	yes	

TB-02-0090	58	1	yes	28	yes	2
TB-02-0095	58	0	yes	31	yes	
TB-02-0098	59	0	yes	70	yes	0
TB-02-0099	59	1	yes	62	yes	
TB-02-0097	60	0	yes	76	yes	2
TB-02-0102	60	1	yes	68	yes	
TB-02-0109	61	1	yes	54	yes	0
TB-02-0110	61	0	yes	60	yes	
TB-02-0111	62	0	yes	53	yes	1
TB-02-0114	62	1	yes	40	yes	
TB-02-0124	63	1	yes	29	yes	4
TB-02-0135	63	0	yes	56	yes	
TB-02-0125	64	1	yes	26	yes	1
TB-02-0131	64	0	yes	22	yes	
TB-02-0132	65	1	yes	22	yes	1
TB-02-0136	65	0	yes	25	yes	
TB-02-0133	66	0	yes	69	yes	4
TB-02-0137	66	1	yes	63	yes	
TB-02-0139	67	1	yes	28	yes	8
TB-02-0143	67	0	yes	46	yes	
TB-02-0140	68	1	yes	25	yes	6
TB-02-0146	68	0	yes	41	yes	
TB-02-0147	69	1	yes	18	yes	13
TB-02-0150	69	0	yes	32	yes	
TB-02-0145	70	0	yes	42	yes	2
TB-02-0149	70	1	yes	42	yes	
TB-02-0151	71	1	yes	48	yes	14
TB-02-0170	71	0	yes	56	yes	
TB-02-0163	72	1	yes	33	yes	14
TB-02-0179	72	0	yes	18	yes	
TB-02-0168	73	1	yes	53	yes	15
TB-02-0186	73	0	yes	43	yes	

TB-02-0169	74	1	yes	20	yes	15
TB-02-0188	74	0	yes	51	yes	
TB-02-0187	75	1	yes	46	yes	7
TB-02-0191	75	0	yes	58	yes	
TB-02-0190	76	0	yes	55	yes	0
TB-02-0192	76	1	yes	31	yes	
TB-02-0196	77	1	yes	87	yes	3
TB-02-0200	77	0	yes	66	yes	
TB-02-0199	78	0	yes	35	yes	0
TB-02-0201	78	1	yes	44	yes	
TB-02-0213	79	1	yes	61	yes	1
TB-02-0215	79	0	yes	79	yes	
TB-02-0228	80	1	yes	58	yes	2
TB-02-0232	80	0	yes	28	yes	
TB-02-0234	81	1	yes	42	yes	4
TB-02-0236	81	0	yes	60	yes	
TB-02-0237	82	0	yes	48	yes	3
TB-02-0241	82	1	yes	28	yes	
TB-02-0242	83	1	yes	26	yes	3
TB-02-0244	83	0	yes	39	yes	
TB-02-0254	84	0	yes	37	yes	1
TB-02-0255	84	1	yes	33	yes	
TB-02-0256	85	1	yes	24	yes	7
TB-02-0267	85	0	yes	52	yes	
TB-02-0258	86	1	yes	21	yes	8
TB-02-0272	86	0	yes	34	yes	
TB-02-0262	87	0	yes	12	yes	2
TB-02-0263	87	1	yes	13	yes	
TB-02-0264	88	0	yes	48	yes	0
TB-02-0265	88	1	yes	22	yes	
TB-02-0266	89	1	yes	63	yes	3
TB-02-0271	89	0	yes	97	yes	

TB-02-0270	90	1	yes	43	yes	8
TB-02-0277	90	0	yes	19	yes	
TB-02-0278	91	1	yes	26	yes	1
TB-02-0279	91	0	yes	56	yes	
TB-02-0280	92	0	yes	60	yes	0
TB-02-0282	92	1	yes	42	yes	
TB-02-0283	93	1	yes	43	yes	2
TB-02-0285	93	0	yes	60	yes	
TB-02-0287	94	0	yes	68	yes	0
TB-02-0288	94	1	yes	71	yes	
TB-02-0247	95	0	yes	15	yes	36
TB-02-0290	95	1	yes	16	yes	
