# Supplementary material for: Diagnostic Accuracy of Molecular Testing on Saliva and Oral Swabs for Pulmonary Tuberculosis
Source: Clin Infect Dis. 2026 Mar 12;82(6):e1313–21. doi: 10.1093/cid/ciag055 (PMC13341261; doi:10.1093/cid/ciag055)
Supplement: ciag055_Supplementary_Data [file ciag055_supplementary_data.zip › Supplementary results.pdf]

Supplementary results

- **Table S1. Sensitivity and specificity of saliva and oral swab samples for the diagnosis of pulmonary tuberculosis by subgroup** 2
- **Discomfort and Acceptability** 3
  - **Table S2.** Acceptability 5-point Likert scale 3
- **Figure S1. Specificity of saliva and oral swab samples for the diagnosis of pulmonary tuberculosis, by subgroup** 4

Table S1. Sensitivity and specificity of saliva and oral swab samples for the diagnosis of pulmonary tuberculosis by subgroup

Index test	Xpert Ultra on Saliva									Xpert Ultra on Oral swab							
**Subgroup**	**Total**	**TP**	**FN**	**TN**	**FP**	**Sensitivity (95% CI)**	***p****	**Specificity (95% CI)**	***p****	**TP**	**FN**	**TN**	**FP**	**Sensitivity (95% CI)**	***p****	**Specificity (95% CI)**	***p****
**Smear microscopy**							**<0.001**		1.0						**<0.001**		NA
Positive	70	69	0	1	0	100% (94.8-100)		100% (2.5-100)		60	9	1	0	87.0% (76.7-93.9)		100% (2.5-100)	
Negative	103	15	9	76	3	62.5% (40.6-81.2)		96.2% (89.3-99.2)		7	17	79	0	29.2% (12.6-51.1)		100% (95.4-100)	
**PLHIV**							0.56		0.08						0.039		1.0
Yes	20	7	1	10	2	87.5% (47.3-99.7)		83.3% (51.6-97.9)		3	5	12	0	37.5% (8.5-75.5)		100% (73.5-100)	
No	170	79	8	81	2	90.8% (82.7-95.9)		97.6% (91.6-99.7)		65	22	82	1	74.7% (64.3-83.4)		98.8% (93.5-100)	
**History of TB**							1.0		0.27						0.67		1.0
Yes	13	6	0	6	1	100% (54.1-100)		85.7% (42.1-99.6)		5	1	7	0	83.3% (35.9-99.6)		100% (59.0-100)	
No	177	80	9	85	3	89.9% (81.7-95.3)		96.6% (90.4-99.3)		63	26	87	1	70.8% (60.2-79.9)		98.9% (93.8-100)	
**Cough ≥2 weeks' duration**							0.20		0.27						0.27		1.0
Yes	174	79	7	85	3	91.9% (83.9-96.7)		96.6% (90.4-99.3)		63	23	87	1	73.3% (62.6-82.2)		98.9% (93.8-100)	
No	16	7	2	6	1	77.8% (40.0-97.2)		85.7% (42.1-99.6)		5	4	7	0	55.6% (21.2-86.3)		100% (59.0-100)	
**Samples collected**							0.73		0.64						0.82		1.0
Before expectoration	109	47	6	53	3	88.7% (77.0-95.7)		94.6% (85.1-98.9)		37	16	55	1	69.8% (55.7-81.7)		98.2% (90.4-100)	
After expectoration	81	39	3	38	1	92.9% (80.5-98.5)		97.4% (86.5-99.9)		31	11	39	0	73.8% (58.0-86.1)		100% (91.0-100)	
**Sex**							0.20		0.57						0.41		1.0
Female	42	21	0	21	0	100% (83.9-100)		100% (83.9-100)		17	4	21	0	81.0% (58.1-94.6)		100% (83.9-100)	
Male	148	65	9	70	4	87.8% (78.2-94.3)		94.6% (86.7-98.5)		51	23	73	1	68.9% (57.1-79.2)		98.6% (92.7-100)	
**Healthcare setting**							0.73		0.61						0.65		1.0
Inpatient	77	39	5	31	2	88.6% (75.4-96.2)		93.9% (79.8-99.3)		33	11	33	0	75.0% (59.7-86.8)		100% (89.4-100)	
Outpatient	113	47	4	60	2	92.2% (81.1-97.8)		96.8% (88.8-99.6)		35	16	61	1	68.6% (54.1-80.9)		98.4% (91.3-100)	
**Sputum induction**							0.17		1.0						0.68		1.0
Yes	24	6	2	16	0	75.0% (34.9-96.8)		100% (79.4-100)		5	3	16	0	62.5% (24.5-91.5)		100% (79.4-100)	
No	166	80	7	75	4	92.0% (84.1-96.87)		94.9% (87.5-98.6)		63	24	78	1	72.4% (61.8-81.5)		98.7% (93.1-100)	

*Fisher-exact test (two-sided *p*-value), a *p*-value <0.0031 was considered significant after Bonferroni correction for multiple hypothesis testing.

Abbreviations: TB: tuberculosis, PLHIV: people living with HIV, TP: True positive, FN: False negative, TN: True negative, FP: False positive, CI: confidence interval.

Discomfort and Acceptability

Participants

A total of 172 participants agreed to respond between February 21 and August 30, 2024; one was excluded for not completing all questions. Responses from 171 participants were analyzed (median age 53 years, IQR 33–66): 88 in the saliva group (53% male; median age 56, IQR 36–66) and 83 in the oral-swab group (53% male; median age 48, IQR 31–66).

Discomfort

Most participants reported no discomfort: 76% (67/88) and 82% (68/83) in the saliva and oral swab groups respectively indicated no discomfort. Only 17% (15/88) in the saliva group and 7% (6/83) in the oral swab group scored at least moderate perceived discomfort (≥5). The median discomfort score was 0 (IQR 0–0) in both groups; no significant difference was found between sampling methods (U=7828.5; *p*=0.25). Similarly, median discomfort did not differ by sex (U=7649.5; p=0.44), and Spearman's rank correlation between age and discomfort was not significant (rho=-0.08; *p*=0.25).

Acceptability

Sample collection was highly acceptable for both saliva and oral-swab methods, with more than 95% of participants selecting 'Somewhat agree' or 'Strongly agree' for each item (**Table S2**). Cronbach's alpha for the three acceptability questions was 0.92, indicating excellent internal consistency. Median acceptability score (ranging from 1 = strongly disagree to 5 = strongly agree) was 5.0 (IQR 4.5–5.0) in the saliva group and 5.0 (IQR 4.0–5.0) in the oral-swab group; by sex, medians were 5.0 (IQR 4.67–5.0) for females and 5.0 (IQR 4.33–5.0) for males. Mann–Whitney U tests showed no significant difference in median acceptability score between saliva versus oral swab (U=7644, *p*=0.77) or between females versus males (U=8022, *p*=0.45). Spearman's correlation between acceptability score and age was non-significant (rho=-0.02; *p*=0.76). However, the correlation between acceptability score and discomfort score was significant and negative (rho=-0.24; *p*=0.001), indicating that higher discomfort scores were associated with slightly lower acceptability score.

The quantile regression of the acceptability score on age, discomfort score, sample type, and sex showed that only the discomfort score was associated significantly: each one-point increase in discomfort score was associated with a 0.0370-point decrease in the median acceptability score (95% CI–0.05 to –0.02; *p*<0.001). Age, sample type, and sex had no effect (all, *p*=1.0).

Table S2. Acceptability 5-point Likert scale

Acceptability	Strongly disagree, n (%)	Somewhat disagree, n (%)	Neutral, n (%)	Somewhat agree, n (%)	Strongly agree, n (%)
**The sample collection seems acceptable to me**					
Saliva	0 (0.0)	0 (0.0)	0 (0.0)	21 (23.9)	67 (76.1)
Oral swab	0 (0.0)	0 (0.0)	0 (0.0)	24 (28.9)	59 (71.1)
**I think the sample collection is good**					
Saliva	0 (0.0)	0 (0.0)	2 (2,3)	20 (22.7)	66 (75.0)
Oral swab	0 (0.0)	0 (0.0)	2 (2,4)	23 (27.7)	58 (69.9)
**I think the sample collection is tolerable**					
Saliva	0 (0.0)	0 (0.0)	0 (0.0)	23 (26.1)	65 (73.9)
Oral swab	0 (0.0)	0 (0.0)	0 (0.0)	23 (27.7)	60 (72.3)

Figure S1. Specificity of saliva and oral swab samples for the diagnosis of pulmonary tuberculosis, by subgroup

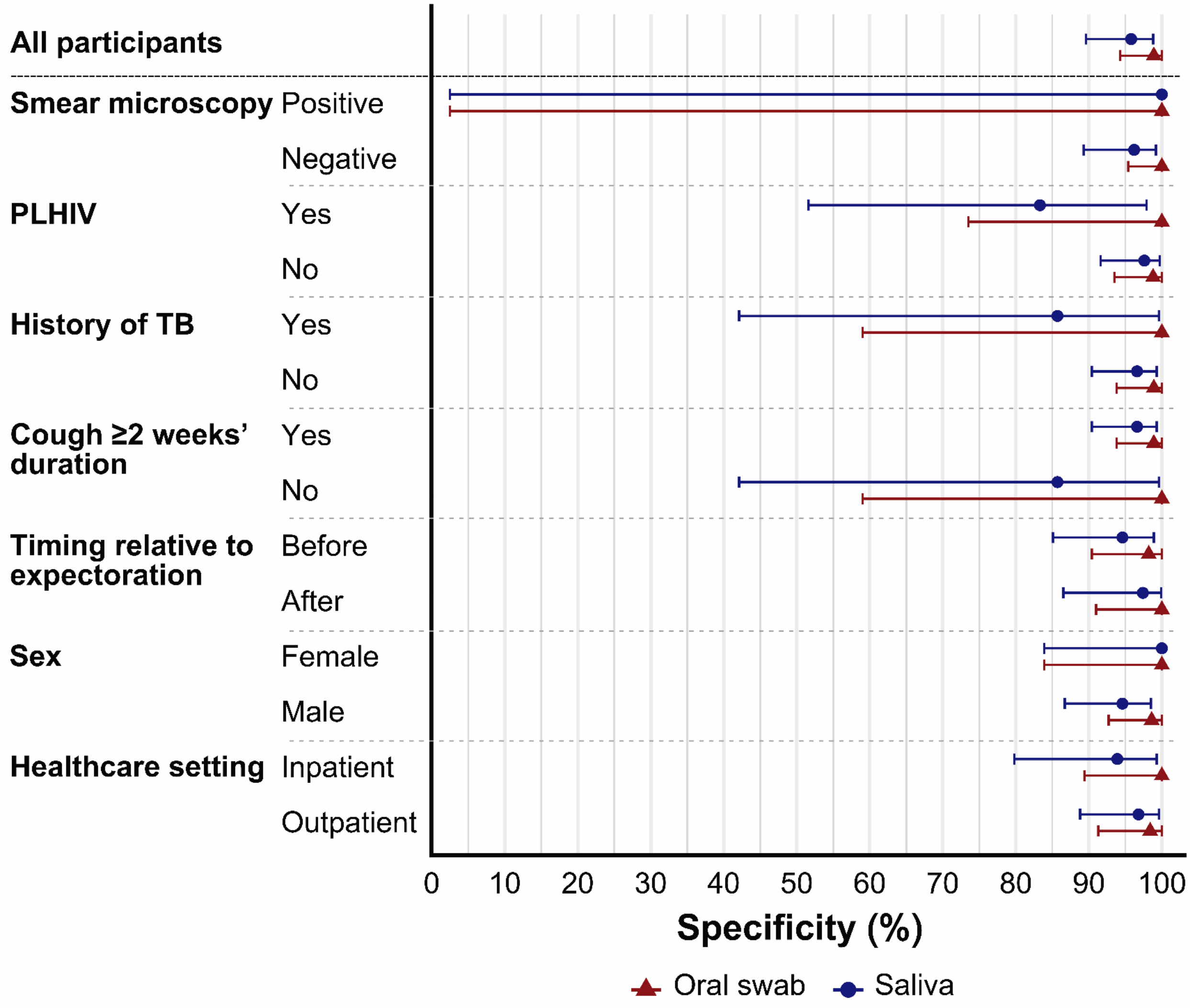

Legend: Specificity of saliva and oral swab samples for the diagnosis of pulmonary tuberculosis. Forest plot showing the specificity with 95% confidence intervals for each sample type, oral swab (red) and saliva (blue), across different participant subgroups, including: smear microscopy status, HIV status, history of TB, cough duration, Timing relative to expectoration (before or after expectoration), sex and healthcare setting (inpatient vs. outpatient).

Abbreviations: TB: tuberculosis, PLHIV: persons living with HIV.
